# Salivary Electrochemical Cortisol Biosensor Based on Tin Disulfide Nanoflakes

**DOI:** 10.1186/s11671-019-3012-0

**Published:** 2019-06-04

**Authors:** Xinke Liu, Sanford P. C. Hsu, Wai-Ching Liu, Yi-Min Wang, Xinrui Liu, Ching-Shu Lo, Yu-Chien Lin, Sasza Chyntara Nabilla, Zhiwen Li, Yuehua Hong, Chingpo Lin, Yunqian Li, Gang Zhao, Ren-Jei Chung

**Affiliations:** 1 College of Materials Science and Engineering, Shenzhen University, No. 3688, Nanhai Ave, Shenzhen, 518060 China; 2 Department of Neurosurgery, Neurological Institute, Taipei Veterans General Hospital, Taipei, 11217 Taiwan; 3 School of Medicine, National Yang Ming University, Taipei, 11221 Taiwan; 4 Department of Chemical Engineering and Biotechnology, National Taipei University of Technology, No. 1, Sec. 3, Zhongxiao E. Rd, Taipei, 10608 Taiwan; 5 Department of Neurosurgical Oncology, First Hospital, Jilin University, Changchun, 130021 China

**Keywords:** Cortisol, 2D Tin disulfide nanoflakes, Electrochemical biosensor, Enzyme-linked immunosorbent assay

## Abstract

Cortisol, a steroid hormone, is secreted by the hypothalamic-pituitary-adrenal system. It is a well-known biomarker of psychological stress and is hence known as the “stress hormone.” If cortisol overexpression is prolonged and repeated, dysfunction in the regulation of cortisol eventually occurs. Therefore, a rapid point-of-care assay to detect cortisol is needed. Salivary cortisol electrochemical analysis is a non-invasive method that is potentially useful in enabling rapid measurement of cortisol levels. In this study, multilayer films containing two-dimensional tin disulfide nanoflakes, cortisol antibody (C-M_ab_), and bovine serum albumin (BSA) were prepared on glassy carbon electrodes (GCE) as BSA/C-M_ab_/SnS_2_/GCE, and characterized using electrochemical impedance spectroscopy and cyclic voltammetry. Electrochemical responses of the biosensor as a function of cortisol concentrations were determined using cyclic voltammetry and differential pulse voltammetry. This cortisol biosensor exhibited a detection range from 100 pM to 100 μM, a detection limit of 100 pM, and a sensitivity of 0.0103 mA/Mcm^2^ (*R*^2^ = 0.9979). Finally, cortisol concentrations in authentic saliva samples obtained using the developed electrochemical system correlated well with results obtained using enzyme-linked immunosorbent assays. This biosensor was successfully prepared and used for the electrochemical detection of salivary cortisol over physiological ranges, based on the specificity of antibody-antigen interactions.

## Introduction

Cortisol, a steroid hormone, is secreted by the hypothalamic-pituitary-adrenal (HPA) system. It is a well-known biomarker of psychological stress and hence called the “stress hormone” [1, 2]. Cortisol levels follow a circadian rhythm over a 24-h cycle; the highest levels are observed early morning, and the levels progressively reduce by night [3–6]. Excessive levels of cortisol can cause Cushing’s disease, with symptoms of central obesity, purple striae, and proximal muscle weakness. However, reduced levels of cortisol can lead to Addison’s disease, with chronic fatigue, malaise, anorexia, postural hypotension, and hypoglycemia [7–9]. Therefore, maintaining appropriate cortisol balance is essential for human health.

A growing interest in the measurement of cortisol as a precursor to medically and psychologically relevant events has developed, among which the most recent affliction is post-traumatic stress disorder (PTSD). The importance of aberrant HPA axis function in PTSD is indisputable; hence, traditional assessment methods are still able to provide abundant evidence and information [10–14]. Recently, many studies have reported the importance of cortisol detection and have identified correlations with different illnesses [15–18]. Various studies have confirmed that cortisol is related to autism spectrum disorder [19], depression [20], suicidal ideation [21], childhood adversity, and externalizing disorders [22].

Although identifying cortisol levels represents an important diagnostic tool, routine laboratory cortisol detection techniques such as chromatography [23, 24], radioimmunoassay [25], electro-chemiluminescent immunoassay [26–28], enzyme-linked immunosorbent assay [28, 29], surface plasmon resonance [1, 30, 31], and quartz crystal microbalance [32] involve extensive analysis time, are expensive, and cannot be implemented in point-of-care (POC) settings [33]. Therefore, there is currently a need for sensitive, efficient, and real-time determination of cortisol levels.

In recent years, electrochemical immunoassay methods, which are established on the specific molecular recognition between antigens and antibodies, have emerged as a promising technology due to salient characteristics, such as involving simple devices, rapid analysis, low cost, label-free POC testing, high sensitivity, and low detection thresholds for cortisol in bio-fluids [34, 35]. Electrical potential changes are ascribed to variations in the concentration of electrochemical redox reactions at the electrode. Secreted cortisol eventually enters the circulatory system and can be found in various bio-fluids such as interstitial fluid [36], blood [37], urine [38], sweat [39], and saliva [40]. The advantages of electrochemical detection of salivary cortisol, which is a non-invasive method, with easy sample collection, handling, and storage, have enhanced its potential for application in POC sensors for real-time measurement [41].

An ideal biosensor should have low detection limits, rapid selectivity, and high sensitivity. In order to fabricate an immunosensor, the immobilizing matrix chosen should possess high surface functionality, high biomolecule loading, and low resistance to electron transport, with a high electron transfer rate [42]. However, metal sulfide nanomaterials have been rarely suggested for the immobilization of proteins for electrochemical biosensing. Therefore, here, tin disulfide was selected as a potential immobilizing matrix for immunosensor development in order to detect cortisol present in saliva.

Nano two-dimensional (2D) materials have attracted abundant research interests in the recent decade. There are a variety of kinds of 2D materials ranging from semiconductor to metal and from inorganic to organic [43–46] and related composite [47–50]. The discovery, manufacturing, and investigation on nano 2D material are prevailing streams in various fields. Nano 2D tin disulfide (SnS_2_), an n-type semiconductor with a bandgap of 2.18–2.44 eV [51, 52], consists of Sn atoms sandwiched between two layers of hexagonally disposed and closely arranged sulfur (S) atoms, with adjacent S layers linked by weak van der Waals forces [53]. Because of its intriguing electrical properties, high carrier mobility, good chemical stability, low cost, and optical properties [54], SnS_2_ has evolved into a promising material for various applications in solar cells and optoelectronic devices [55, 56], as electrodes in lithium-ion batteries [57, 58], gas sensors, and glucose monitors [59, 60]. The selection of electrode material is an important key factor to improve the performance by providing a large reaction area and favorable microenvironment for facilitating electron transfer between enzyme and electrode surface.

In this work, biosensors were fabricated using SnS_2_ as the immobilizing matrix to detect cortisol. The results of differential pulse voltammetry (DPV) studies related to electrochemical sensing show a high sensitivity of 0.0103 mA/Mcm^2^ and the lowest detection concentration of 100 pM.

## Materials and Methods

### Materials

Hydrocortisone (cortisol), anti-rabbit cortisol antibody (anti-cortisol, C-M_ab_), potassium hexacyanoferrate (II), potassium hexacyanoferrate (III), β-estradiol, testosterone, progesterone, and corticosterone were purchased from Sigma-Aldrich (St. Louis, MO, USA). Bovine serum albumin (BSA) was obtained from PanReac. Tin (IV) chloride pentahydrate (SnCl_4_^**.**^5H_2_O) and thioacetamide (C_2_H_5_NS) were supplied by Showa (Japan) and Alfa Aesar (UK). Phosphate buffered saline (PBS) prepared with NaCl, KCl, Na_2_HPO_4_, and KH_2_PO_4_ were purchased from Sigma-Aldrich. Micro-polished alumina was sourced from Buehler (UK). All other chemicals were of analytical grade and were used without further purification. Cortisol Saliva ELISA kit (Cat # SA E-6000) was purchased from LDN (Germany).

### Synthesis of Tin Disulfide

Powders of SnCl_4_·5H_2_O and C_2_H_5_NS were mixed in 70 mL deionized water and adjusted pH to 7.4. A hydrothermal autoclave reactor containing the reactants was heated from room temperature to 200 ^°^C in 1 h, and maintained at 200 ^°^C for 11 h. Then, the resulting SnS_2_ powder was washed with deionized water and ethanol at 6000 rpm for 15 min, and finally dried in air at 80 ^°^C. This hydrothermal method was successfully applied for the synthesis of SnS_2_.

### Materials Characterization

X-ray diffraction (XRD, PANalytical, The Netherlands) was utilized to investigate the crystal phase of 2D hexagonal SnS_2_ flakes. Multi-functional field emission scanning electron microscopy (FE-SEM, Zeiss, Germany) was used to image the surface morphology of materials. Field emission gun transmission electron microscopy (FEG-TEM, Tecnai, USA) was used to discern the microstructure of SnS_2_, and selected area diffraction (SAED, Tecnai) was used to obtain crystal patterns.

### Fabrication of BSA/C-M_ab_/SnS_2_/GCE Biosensors

**Fig. 1:**
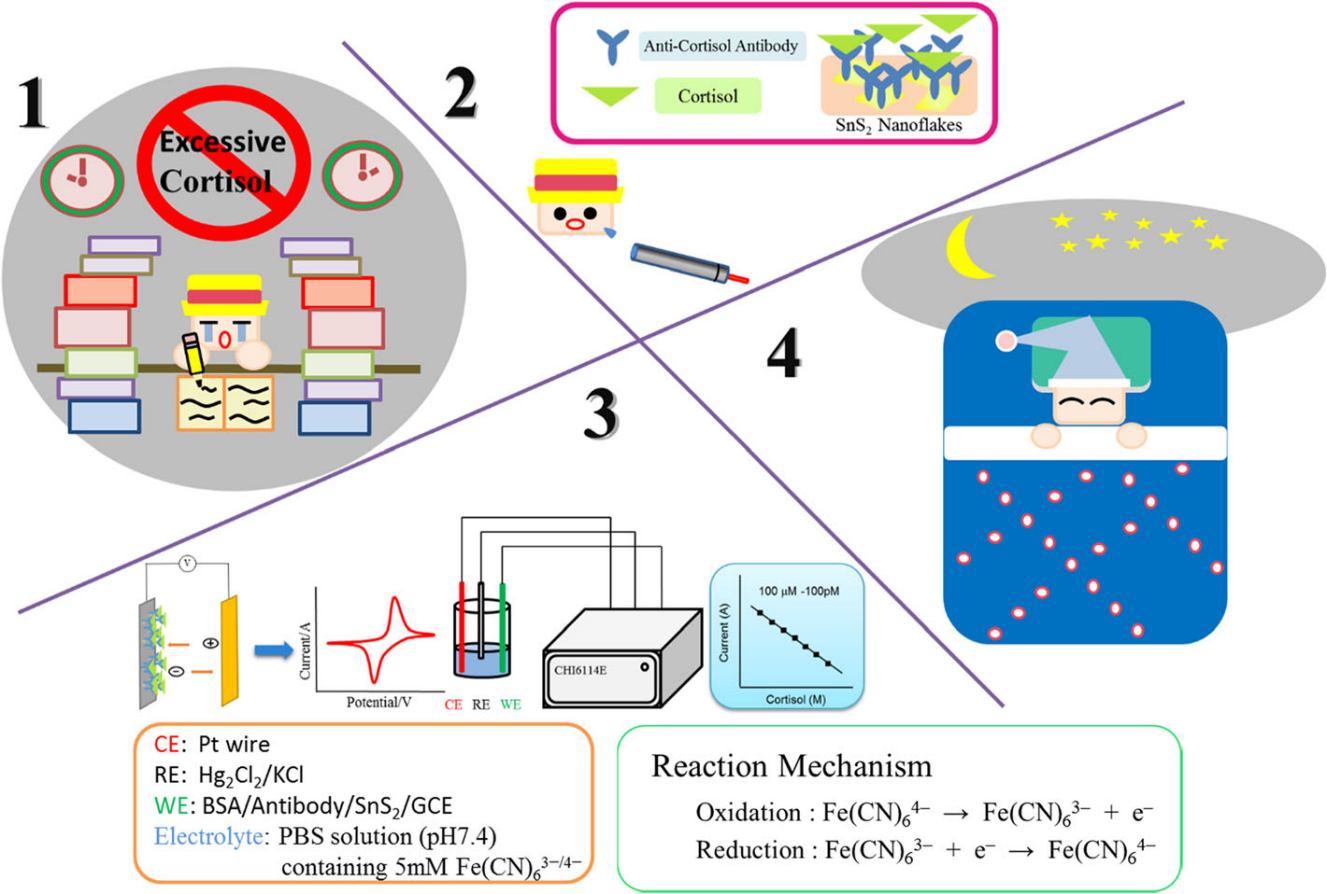
Research concept and setup of the detection system

Glassy carbon electrodes (GCEs) were first polished with alumina slurry, and then drops of a mixture of 5 M SnS_2_ were deposited on the surface of pretreated GCEs. Solutions of anti-cortisol antibody (1 mg/mL) and BSA (1%) were prepared in PBS. SnS_2_/GCE was then decorated with the antibody and BSA solutions in sequence. The fabricated BSA/C-M_ab_/SnS_2_/GCE biosensors were stored under refrigeration at 4 ^°^C when not in use. The research concept and setup of detection system are illustrated in Fig. 1.

### Electrochemical Analysis

Fabricated BSA/C-M_ab_/SnS_2_/GCEs were characterized using electrochemical impedance spectroscopy (EIS) and cyclic voltammetry (CV) to compare their electro-active behaviors. Electrochemical response studies as a function of cortisol concentration were conducted using CV and differential pulse voltammetry (DPV). All the experiments were performed using a three-electrode system with a GCE as the working electrode, a Pt wire as the auxiliary electrode, and a saturated calomel electrode as the reference electrode in 10 mM PBS (pH 7.4) containing 5 mM Fe(CN)_6_^3-/4-^. Electrochemical measurements were performed on a Model CHI6114E series electrochemical workstation (CH Instruments, USA). The CV and DPV measurements were carried out between − 0.4 V and 1.0 V at 10 mV/s scan rate, unless specified otherwise.

### Saliva Sample Collection and Electrochemical Sensing

Saliva sample (2 mL) was collected from two healthy voluntary subjects at around noon for validating the developed BSA/C-M_ab_/SnS_2_/GCE. Saliva samples were obtained without any filtration and initially stored at − 20 °C for maintaining biological characteristics. Before sensing, the saliva samples were thawed to room temperature and centrifuged at 3500 rpm for 15 min to collect the supernatant for measurement. The separated saliva was stored at − 20 °C. The BSA/C-M_ab_/SnS_2_/GCE was utilized for the electrochemical sensing of cortisol concentrations in saliva samples. The detection of cortisol using electrochemical analysis with the BSA/C-M_ab_/SnS_2_/GCE was compared with that of the commercially available ELISA cortisol kit mentioned above.

### Interference Study

The inhibitory effect of potential confounding agents, such as other steroid hormones, on BSA/C-M_ab_/SnS_2_/GCE specificity was investigated by placing the biosensor in the following different solutions: 100 nM β-estradiol, 100 nM testosterone, 100 nM progesterone, and 100 nM corticosterone, for 10 min and then scanned by CV. The scanning rate was 10 mV/s and the scanning range was from − 0.4 V to 0.6 V.

### Detection of Salivary Cortisol by ELISA

ELISA was performed on the saliva samples according to the manufacturer’s protocol. To establish a calibration curve for cortisol measurements, the assay was performed in a 96-well titer plate containing six known standard cortisol concentrations (0.0, 0.1, 0.4, 1.7, 7.0, and 30 ng/mL) for determining the absorbance of each well at 450 nm. The calibration curve was fitted with a trendline to obtain an equation for the calculation of unknown samples.

## Results and Discussion

### Material Analysis of SnS_2_

**Fig. 2:**
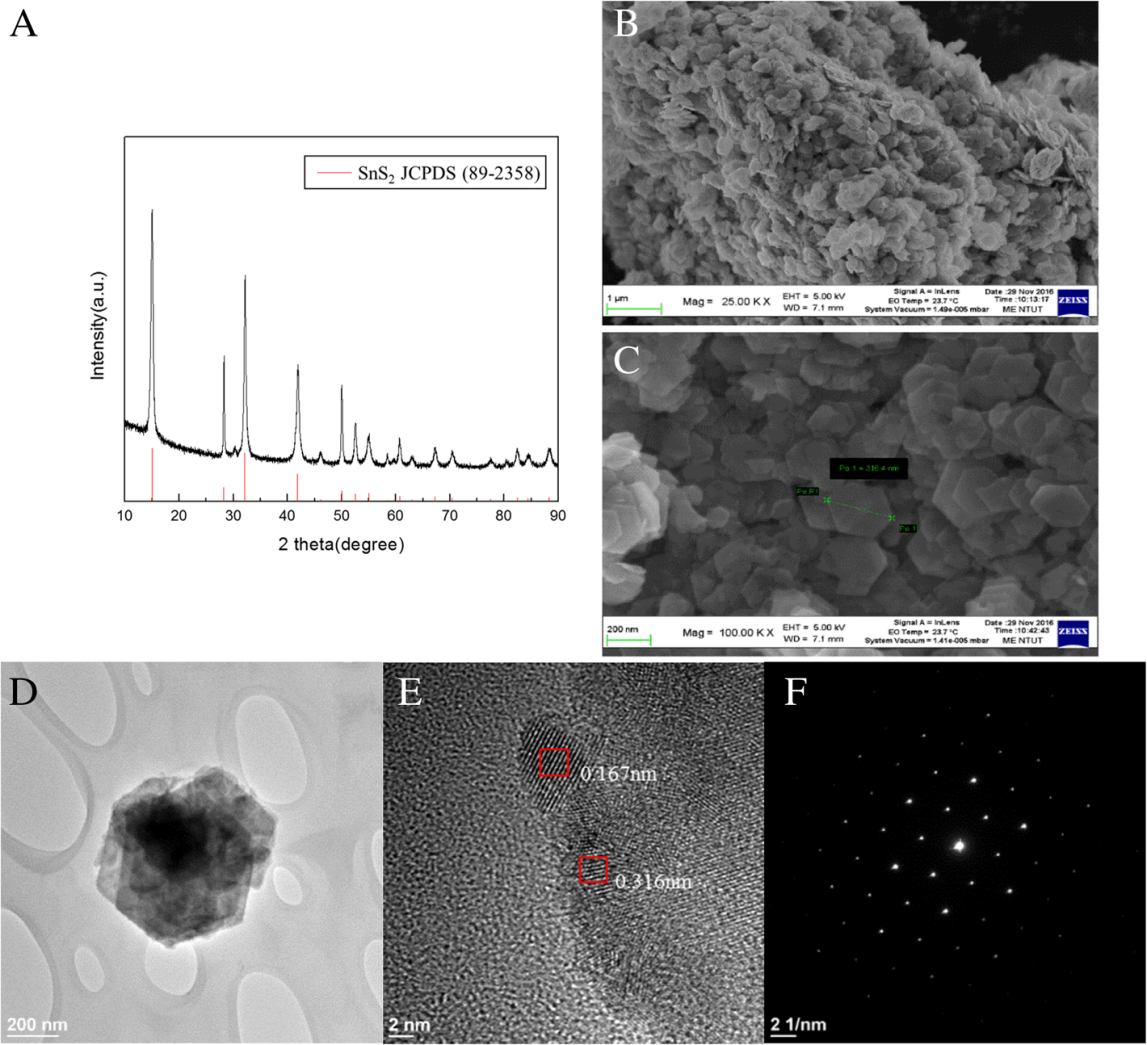
**a** XRD pattern of SnS_2_. FE-SEM images of SnS_2_ nanoflakes were taken at magnifications of (**b**) × 250,000 and (**c**) × 100,000. **d** FEG-TEM images of SnS_2_ nanoflakes. **e** Cross-sectional FEG-TEM of SnS_2_ nanoflakes and zoomed-in FEG-TEM image. **f** SAED image of SnS_2_ nanoflakes

As seen from the XRD pattern in Fig. 2a, the as-synthesized product displays only the XRD peaks corresponding to the hexagonal phase SnS_2_ (JCPDS card no. 89-2358). Figure 2b, c illustrates the FE-SEM images of the as-synthesized SnS_2_ having uniform flake-like morphology with a size of approximately 300 nm. Figure 2d–f shows the FEG-TEM and SAED images of SnS_2_, in which lattice fringe spacings of 0.167 nm and 0.316 nm are identified for hexagonal SnS_2_ as a single crystalline structure. The stacking of nanoflakes is less than 10 layers with a total thickness of less than 10 nm.

### Electrochemical Responses of the Electrode

**Fig. 3:**
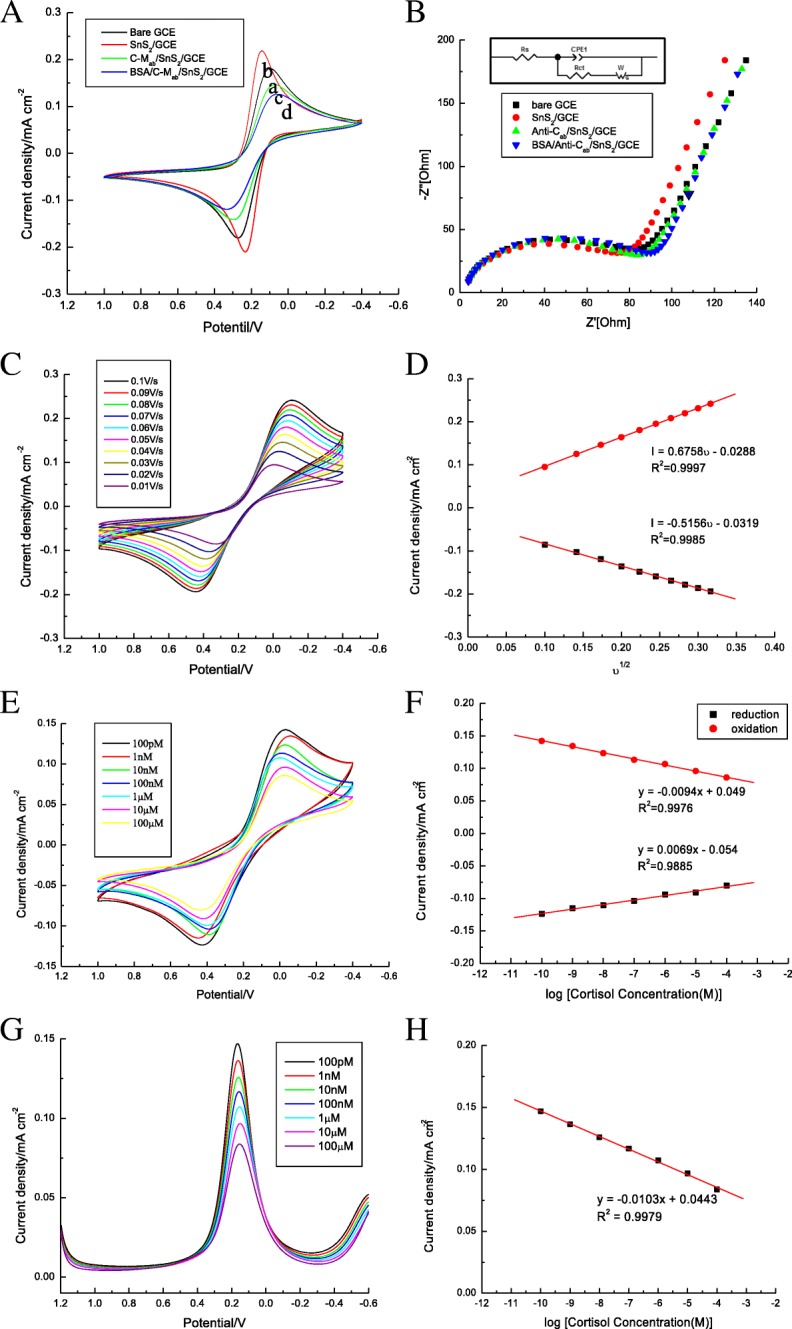
**a** CV response study of GCE electrode (curve a), SnS_2_/GCE electrode (curve b), C-M_ab_/SnS_2_/GCE electrode (curve c), BSA/C-M_ab_/SnS_2_/GCE electrode (curve d). **b** EIS response study of the GCE, SnS_2_/GCE, C-M_ab_/SnS_2_/GCE, and BSA/C-M_ab_/SnS_2_/GCE electrodes. Inset: the corresponding equivalent circuit. **c** Increased magnitude of oxidation response current of BSA/C-M_ab_/SnS_2_/GCE electrode with increased scan rate from 10 mV/s to 100 mV/s. **d** The current magnitude increased with increasing scan rate. **e** CV studies of BSA/C-M_ab_/SnS_2_/GCE electrode as a function of cortisol concentration varying from 100 pM to 100 μM. **f** Linearity curve for the current response with different cortisol concentrations. **g** DPV studies of BSA/C-M_ab_/SnS_2_/GCE electrode as a function of cortisol concentration varying from 100 pM to 100 μM. **h** Linearity curve for the current response with different cortisol concentrations

Oxidation current can greatly increase by the addition of tin disulfide. As shown in Fig. 3a, b, the magnitude of the oxidation current reduced from SnS_2_/GCE to C-M_ab_/SnS_2_/GCE, followed by BSA/C-M_ab_/SnS_2_/GCE, as the charge transfer resistance value increased. Therefore, the results indicate that the sensor properties were modified on the electrode. Initially, BSA/C-M_ab_/SnS_2_/GCE was studied by varying the scan rate from 10 mV/s to 100 mV/s, as shown in Fig. 3c. The change in current response with scan rate, as plotted in Fig. 3d, shows that the oxidation current increased linearly with scan rate, and followed the relation: *I* = 0.5156 υ–0.0319 (*R*^2^ = 0.9985) in oxidation, and *I* = 0.6758υ–0.0288 (*R*^2^ = 0.9997) in reduction. However, near-linearity for the increment in peak current with increasing scan rate with well-defined redox peaks indicates a surface-controlled process, with stable electron transfer.

The current decreased with increasing concentration of cortisol over the range of 100 pM to 100 μM. The difference in current directly correlated to the cortisol concentration being sensed. Current values and well-separated oxidation peaks were obtained for BSA/C-M_ab_/SnS_2_/GCE electrodes, as shown in Fig. 3e, f. The change in current with the log of concentration was nearly linear. It is clear that the reduction in the linear regression coefficient is better for CV. Therefore, further measurements were made with more specific and accurate DPV. The results of such DPV studies indicated that the magnitude of current response decreased with the addition of cortisol, as illustrated in Fig. 3g. A calibration curve presented in Fig. 3h plots the magnitude of current response and logarithm of cortisol concentration, and was found to be linearly dependent and to follow the equation: *y* = − 0.0103*x* + 0.0443; *R*^2^ = 0.9979. This sensor exhibited a detection range between 100 pM to 100 μM, with a limit of detection of 100 pM and a sensitivity of 0.0103 mA/Mcm^2^ (*R*^2^ = 0.9979).

### Storage Stability Study

**Fig. 4:**
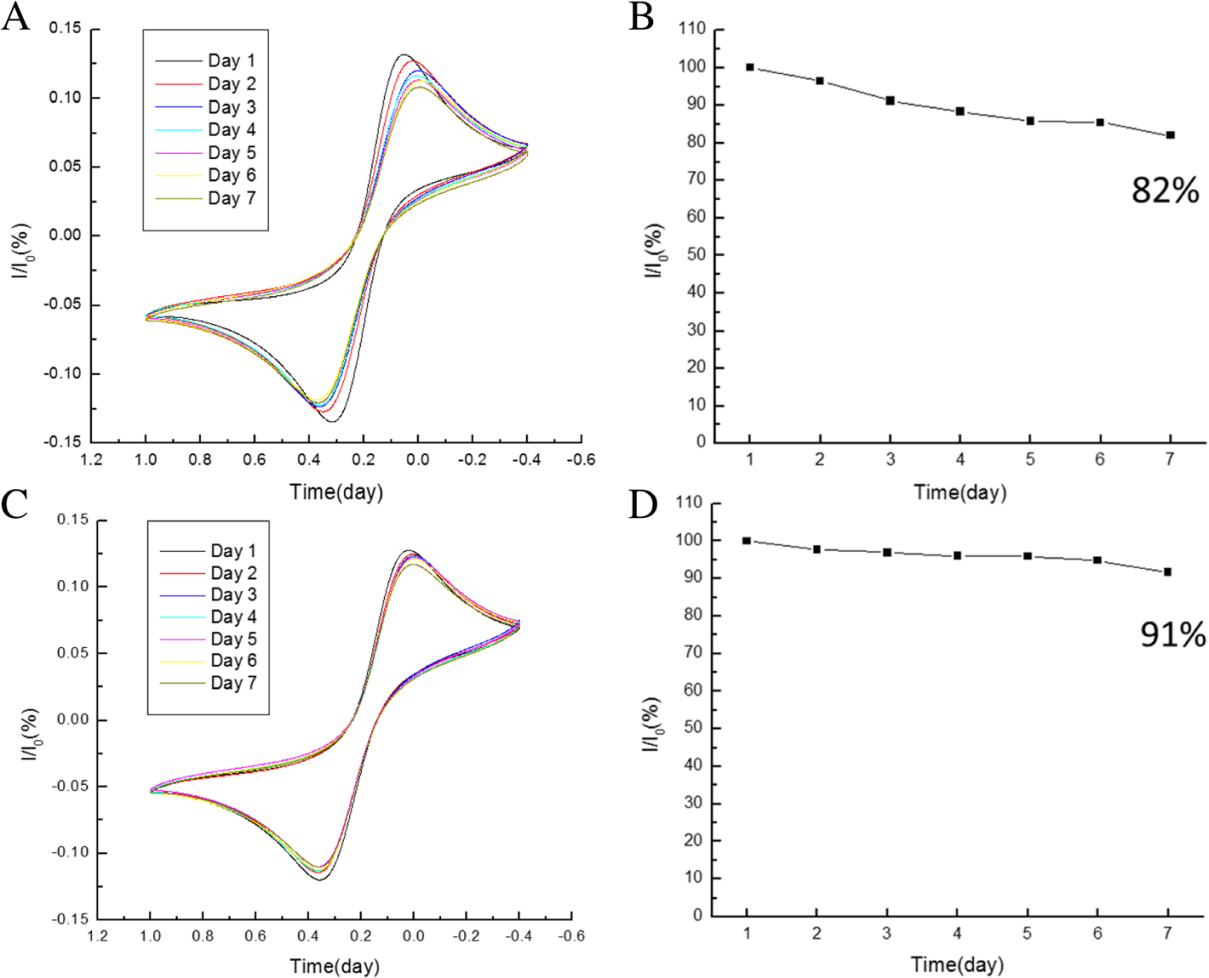
Redox peak stability of BSA/C-M_ab_/SnS_2_/GCE electrode with different preservation conditions (**a** and **b**) under vacuum (**c** and **d**) at 4 °C for 7 days

CV studies were also carried out to study the shelf life of the BSA/C-M_ab_/SnS_2_/GCE at intervals of 1 day to 1 week. In order to compare two preservation conditions, one condition was to store the electrodes dried under vacuum, while the other was to store the electrodes at 4 °C. The redox peak stability of the electrodes at 4 °C and under vacuum are shown in Fig. 4a, c, respectively. It is clear that the preservation condition at 4 °C was better than that under vacuum. Figure 4b, d shows that the electrode stability value was 82% with the electrodes stored under vacuum for 7 days, while the electrode stability value was 91% with the electrodes stored at 4 °C. It can be observed that the stability of electrodes stored at 4 °C was higher than that under vacuum. The loss of activity of the electrode was possibly caused by degradation of the cortisol antibody activity under vacuum. The storage stability is a crucial issue for enzymatic sensor. A protective coating may be introduced in the future design of the electrode.

### Interference Study

**Fig. 5:**
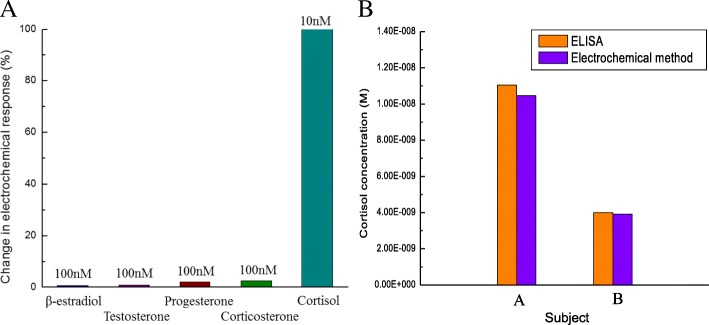
**a** Interference study involving β-estradiol (100 nM), testosterone (100 nM), progesterone (100 nM), and corticosterone (100 nM) with respect to cortisol (1 0nM). **b** Comparison of salivary cortisol measurements using ELISA and electrochemical methods

The results of CV studies of BSA/C-M_ab_/SnS_2_/GCE for measuring potential confounding agents, such as β-estradiol (100 nM), testosterone (100 nM), progesterone (100 nM), and corticosterone (100 nM) with respect to cortisol (10 nM), are shown in Fig. 5a. Compared to the change in the response of the cortisol signal, the effects of interference were less than 5% of the result for cortisol, suggesting that such potential interferences can be conveniently neglected.

### Detection of Salivary Cortisol Using ELISA and Electrochemical Methods

Measurements of salivary cortisol samples performed with ELISA and the BSA/C-M_ab_/SnS_2_/GCE electrode are summarized in Table 1 and Fig. 5b. The concentrations of cortisol determined using ELISA were 1.105 ×10^−8^ M and 3.998 × 10^−9^ M. The calculated results of cortisol using electrochemical measurement were 1.046 × 10^−8^ M and 3.911 × 10^−9^ M. Good correlation was achieved with these two techniques, exhibiting comparable results with only a 2–5% difference. Hence, the results demonstrate that this BSA/C-M_ab_/SnS_2_/GCE can be employed for electrochemical cortisol sensing in biologically relevant fluids such as saliva.

**Table 1:** Measurements of cortisol concentration in authentic saliva samples using ELISA and our developed electrochemical method

Subject	Saliva collection time	Calculated cortisol concentration (M)	
		ELISA	Electrochemical method
A	12:48 PM	1.105 × 10^−8^	1.046 × 10^−8^
B	1:30 PM	3.998 × 10^−9^	3.911 × 10^−9^

### Comparison with Other Studies

The results of this study were compared with other studies involving electrochemical sensors of salivary cortisol reported in the literature in order to gain a better understanding of the performance of this BSA/C-M_ab_/SnS_2_/GCE. Tables 2 and 3 show comparisons of results obtained using non-gold electrodes in cortisol detection. There are three main advantages of the present work. First, the materials are much lower in cost than the devices presented in other studies. Second, the preparation process was relatively simple and rapid. Finally, the detection limit was similar to that reported in other literature or, indeed, even better than those reported, while the target detection range for salivary cortisol is easily obtained.

**Table 2:** Comparisons of modified non-gold electrodes to the cortisol detection results reported in the literature and in the present study

Substrate	Detection limit (ng/mL)	Sensitivity	Sample	Technique	Reference
Surface plasma resonance (SPR) biosensor	1.0	_	Saliva	SPR	[1]
Screen printed carbon electrode	0.0035	_	Serum	DPV	[61]
Pt electrode	1.0	200 nA (200 mg dL^−1^)^−1^	Saliva	Current by GOD cortisol reaction	[62]
HRP-strept-biotin-Ab-Cor/AuNPs/MrGO/Nafion@GCE	0.05	8.2443 μA ng^−1^ mL^−1^	Blood	DPV	[63]
*BSA/anti-C*_*ab*_ */SnS*_*2*_ */GCE*	*0.036*	*0.0103 mA* ^*−1*^ *c* ^*−2*^	*Saliva*	*DPV*	*Current study*

**Table 3:** Comparisons of modified gold electrode and the cortisol detection results reported in the literature and in the present study

Substrate	Detection limit (ng/mL)	Sensitivity	Sample	Technique	Reference
Au IDmEs	0.00036	3.2 kΩ (pg mL^−1^)^−1^	Saliva/ISF	EIS	[64]
Au IDmEs	0.00036	7.9 kΩ (pg mL^−1^)^−1^	Saliva	EIS	[65]
Au IDmEs	0.00036	6.4 kΩ (pg mL^−1^)^−1^	ISF	EIS	[12]
PANI protected Au Nanoparticles/Au IDmEs	0.00036	4.5 μA (g mL^−1^)^−1^	Cortisol in PBS solution	CV, DPV	[34]
Au nanoparticle/Au IDmEs	0.016	1.6 μA (pg mL^−1^)^−1^	Blood	Square wave voltammetry	[66]
Reduced graphene (rGo)/Au IDA	1.0	_	Saliva	CV	[67]
Core-shell Ag@AgO-PANI/Au IDmEs	0.00064	183 μA (g mL^−1^)^−1^	Cortisol in PBS solution	CV	[68]
Au IDmEs	0.01	6 μA (pg mL^−1^)^−1^	Saliva	CV	[6]
*BSA/anti-C*_*ab*_ */SnS*_*2*_ */GCE*	*0.036*	*0.0103 mAM* ^*−1*^ *cm* ^*−2*^	*Saliva*	*DPV*	*Current study*

## Conclusions

A hydrothermal method has been successfully applied for the synthesis of SnS_2_. The properties of SnS_2_ were characterized by XRD, FE-SEM, FEG-TEM, and SAED. Electrochemical responses of the electrode as a function of cortisol concentrations were determined using CV and DPV. Our cortisol sensor exhibited a detection range from 100 pM to 100 μM, a detection limit of 100 pM, and sensitivity of 0.0103 mA/Mcm^2^ (*R*^2^ = 0.9979). The obtained sensing parameters were in normal physiological ranges. The impact of potential interference was less than 5%, indicating good specificity of this sensor. Stability testing demonstrated that the activity of the sensor stored at 4 °C was better than under vacuum. The results of this electrode for the measurement of cortisol in saliva samples were consistent with ELISA. Therefore, electrochemical analysis using this BSA/C-M_ab_/SnS_2_/GCE electrode can replace more traditional time-consuming immunoassay approaches.

## Data Availability

All data generated or analyzed during this study are included in this published article.

## Abbreviations

2D: Two-dimensional
BSA: Bovine serum albumin
C-M_ab_: Cortisol antibody
CV: Cyclic voltammetry
DPV: Differential pulse voltammetry
EIS: Electrochemical impedance spectroscopy
ELISA: Enzyme-linked immunosorbent assay
FEG-TEM: Field emission gun transmission electron microscope
FE-SEM: Field emission scanning electron microscope
GCE: Glassy carbon electrodes
HPA: Hypothalamic-pituitary-adrenal
PBS: Phosphate buffered saline
POC: Point-of-care
PTSD: Post-traumatic stress disorder
SAED: Selected area diffraction
XRD: X-ray diffraction
